# Is It What You Have or What You Do With It? Staffing, Human Resource Management, and Knowledge Management Practices

**DOI:** 10.1093/geroni/igaa057.2364

**Published:** 2020-12-16

**Authors:** Larry Hearld, Akbar Ghiasi, Ganisher Davlyatov, Robert Weech-Maldonado

**Affiliations:** 1 University of Alabama at Birmingham, Birmingham, Alabama, United States; 2 University of Incarnate Word, San Antonio, Texas, United States; 3 UAB, Birmingham, Alabama, United States

## Abstract

Research in the nursing home industry shows more robust knowledge management activities are associated with the adoption of patient-centered, culture change initiatives among high Medicaid nursing homes. These findings are notable because they highlight the important role that knowledge management activities may play for improving quality of care in under-resourced nursing homes. They also raise important questions about the conditions that may support or hinder the use of these activities. Using survey responses from 393 nursing home administrators, we empirically examined whether two components of human resources – staffing levels and HRM practices – are associated with the level of knowledge management activities in high Medicaid census nursing homes. More robust HRM practices were associated with greater levels of knowledge management activities, as well as three separate domains of knowledge management (acquisition, dissemination, and responsiveness). Staffing levels, in contrast, were not significantly associated with the level of knowledge management activities.

